# Morphologic and biochemical changes in the retina and sclera induced by form deprivation high myopia in guinea pigs

**DOI:** 10.1186/s12886-020-01377-1

**Published:** 2020-03-16

**Authors:** Yingxin Zi, Yu Deng, Jingru Zhao, Meiqi Ji, Yali Qin, Tingting Deng, Ming Jin

**Affiliations:** 1 Department of Graduate School, Beijing University of Chinese Medicine, No.11, North 3rd Ring East Road, Chaoyang District, Beijing, 100029 China; 2 Department of Ophthalmology, China-Japan Friendship Hospital, Yinghua Donglu, Chaoyang District, Beijing, 100029 China; 3 Institute of Clinical Medical Sciences, China-Japan Friendship Hospital, Yinghua Donglu, Chaoyang District, Beijing, 100029 China

**Keywords:** Form deprivation, High myopia, Local retinal regulation, Oxygen free radical, Guinea pig

## Abstract

**Background:**

To study the morphologic and biochemical changes in the retina and sclera induced by form deprivation high myopia (FDHM) in guinea pigs and explore the possible mechanisms of FDHM formation.

**Methods:**

Forty 3-week-old guinea pigs were randomized into the blank control (Group I, 20 cases) and model groups (20 cases). In the model group, the right eyes of the guinea pigs were sutured for 8 weeks to induce FDHM (Group II) and the left eyes were considered a self-control group (Group III). The refractive errors were measured with retinoscopy. The anterior chamber depth (AC), lens thickness (L), vitreous chamber depth (V) and axial length (AL) were measured using ultrasonometry A. Retinal and scleral morphology and ultrastructural features were observed with light and electron microscopy. The malondialdehyde (MDA) content and superoxide dismutase (SOD) activity in the retina and sclera were detected with a chemical colorimetric assay.

**Results:**

After 8 weeks of stitching, the refractive errors of Group II changed from (+ 3.59 ± 0.33) D to (− 7.96 ± 0.55) D, and these values were significantly higher than those of Group I (+ 0.89 ± 0.32) D and Group III (− 0.55 ± 0.49) D (*P* < 0.05). The vitreous chamber depth (4.12 ± 0.13) mm and axial length (8.93 ± 0.22) mm of Group II were significantly longer than those of Group I [(3.71 ± 0.23) mm and (7.95 ± 0.37) mm, respectively] and Group III [(3.93 ± 0.04) mm and (8.01 ± 0.15) mm, respectively] (*P* < 0.05). With the prolongation of form deprivation (FD), the retina and scleral tissues showed thinning, the ganglion cell and inner and outer nuclear layers of the retina became decreased, and the arrangement was disordered. In Group II, the SOD activity was significantly lower than that in Group I and Group III; the MDA content was significantly higher than that in Group I and Group III. The differences were statistically significant (*P* < 0.05).

**Conclusions:**

These findings suggested that in the FDHM guinea pigs model, the refractive errors, the vitreous chamber depth, and axial length increased significantly with prolongation of monocular FD time, and morphological structural changes in the retina and sclera were observed. Oxygen free radicals might participate in the formation of FDHM.

## Background

Myopia is a global epidemic ametropia caused by a combination of genetic and environmental factors [1]. Worldwide, the number of people with high myopia (HM) is approximately 163 million, accounting for 2.7% of the total population. It is estimated that by 2050, the number of people with HM will increase to 938 million, accounting for 9.8% of the total population. The number of people with HM in China is 87 million, accounting for 6.3% of the total population. It is estimated that by 2050, the number of people with HM will exceed 175 million, accounting for 13% of the total population [2]. Surveys have shown that the prevalence of HM in Chinese adolescents is 6.69–38.4%; that is, China is a typical country with a high incidence of HM [3, 4]. As myopic refractive errors increase, especially up to HM, the incidence of fundus lesions, risk of blindness and medical costs also increase [5]. Pathological myopia (PM) was reported to be an important cause of global vision loss, and the prevalence of PM is 0.9 to 3.1%. The prevalence of PM associated with visual impairment in European countries is 0.1 to 0.5%, and the prevalence in Asian countries is high, ranging from 0.2 to 1.4%. Studies in China have shown that PM is the most common cause of blindness, accounting for 26.1% of cases [6]. At present, the occurrence of myopia tends to be higher in younger people and is in a more advanced stage. HM fundus lesions, especially macular degeneration, are the main causes of blindness in East Asian countries [7].

The form deprivation myopia (FDM) theory was proposed in 1977. After Wiesel et al. [8] sutured the eyelids of newborn rhesus monkeys, the sutured eyes caused obvious axial myopia. According to research needs, some scholars have carried out myopia research on the eyes of animals such as tree shrews [9, 10], chickens [11], mice [12], rats [13], and guinea pigs [14]. Studies have confirmed that form deprivation (FD) in young animals leads to abnormal growth of the eye axis and the formation of obvious myopia [15, 16]. Guinea pigs are widely used in the current research on myopia since the eyeball structure and emmetropization mechanism are similar to those of human myopia. The mechanism of HM formation is very complicated, and local retinal regulation is a focus of research. It has been shown that in myopic eyes, the axial length increases, and vascular changes, such as retinal vascularization diminishing and narrowing, are observed [17]. Shih’s research revealed that along with the increase in myopic refractive errors, the ocular pulse amplitude, which is generated by choroidal blood flow, was decreased. The circulatory disturbance was observed in the formation of myopia [18]. Oxidative stress (OS) is not only involved in the development of myopia but is also accompanied by the complications of HM. The elevated level of malondialdehyde (MDA) in the vitreous of myopic subjects strongly suggests retinal lipid peroxidation involvement in the genesis of the human myopic cataracts [19]. OS levels and metabolic activities in the aqueous humour were lower in patients with high myopia than the controls [20]. Simonelli et al. [21] demonstrated that lipid peroxidation might be associated with the pathogenesis of severe myopia. MDA, as the breakdown product of lipid peroxidation, would induce the accumulation of soluble proteins and fragmentation of the membrane structure. Bhatia et al. [22] also stated that OS was related to myopic lens. Compared with that in age-related cataracts, the content of superoxide dismutase (SOD) was lower in myopic patients. Compared with that of healthy controls, the MDA level in plasma was higher in myopia. Shkrebets [23] reported that the antioxidant capacity in tears is weakened in HM patients. To some extent, SOD, as the first line of defence against OS and an important component of the enzymatic antioxidant defence system in the retina, can increase the activity of endogenous peroxide detoxifying activity. Xu Huibi et al. [24] reported that the SOD activity decreased in the retina of myopic eyes in form-sense-deprived chicks. However, fewer studies about the activities of total SOD in form deprivation high myopia (FDHM) in guinea pigs have been performed. This study intended to establish a guinea pig model of FDM to simulate HM, observe changes in the retinal and scleral morphology, determine the MDA content in the retina and sclera, and assess the SOD activity. The relationship between oxygen free radicals and the formation of FDHM provides new ideas and a basis for further research on myopia, especially HM.

## Methods

### Animal model and grouping

Forty pigmented guinea pigs at 2 weeks of age weighing 100 g to 140 g were obtained from the Beijing Keyu Animal Breeding Center. {SCXK (Jing) 2017–0002}. After acclimation for 1 week, the guinea pigs were randomly assigned to two groups: the blank control group (Group I) with 20 guinea pigs and the model group with 20 guinea pigs. The model group was divided again into the FDHM group (Group II, eyelids on their right eyes were stitched for 8 weeks) and the self-controlled group (Group III, left eyes received no intervention). The operation of Group II was performed under anesthesia (intraperitoneal injection with 1% pentobarbital sodium, 50 mg/Kg). All animals had access to abundant food and water at the Institute of Chinese Materia Medica, China Academy of Chinese Medical Sciences Central Animal Laboratory {SYXK (Jing) 2016–0013}, and vitamin-rich feed and fresh vegetables were given to supplement vitamin C. This study was approved by the Ethics Committee of the China-Japan Friendship Hospital (ethics review number: 180103) and was conducted in accordance with the Association for Research in Vision and Ophthalmology Statement for the Use of Animals in Ophthalmology and Vision Research and the Declaration of Helsinki. Before the experiment, the anterior segment and fundus of all guinea pig eyes were examined with a slit lamp microscope and an ophthalmoscope, and those with eye diseases were excluded.

### Main equipment and reagents

A strip light inspection mirror was purchased from Suzhou Liuliu Vision Technology Co., Ltd. A lens box was purchased from Danyang Medical Instrument Factory. A type A ophthalmic ultrasound instrument (ODM-1000) was purchased from Tianjin Maida Medical Technology Co., Ltd. Total SOD (KGT001100) and an MDA (KGT004) test kit were purchased from Beijing Benuowei Biotechnology Co., Ltd.

### Biological measurements

All guinea pigs were treated with mydriatic optometry before and after FD for 8 weeks. Before FD, tropicamide eye drops were used to fully enlarge the pupil, and the ciliary muscle was paralyzed. The optometry was performed in a dark room with a band-shaped photoreceptor. Each eye was measured three times to obtain an average value. The astigmatism degree was transformed into half of the count, and this was included in the spherical mirror. Surface anesthesia was performed with 0.4% oxybuprocaine hydrochloride eye drops. The length of each component of the eyeball was measured with ultrasonometry A. The probe of ultrasonometry A was perpendicular to the cornea and located in the center of the pupil. Meanwhile, the cornea was not pressed. When the pattern was stable and clear, the posterior capsule of the lens and the double peak of the retina were higher than the baseline, and the image and results were recorded. Each eye was repeatedly measured 8 times to obtain an average value. The recorded data included the anterior chamber depth (AC), lens thickness (L), vitreous chamber depth (V) and axial length (AL). Biological measurements of all guinea pigs were performed under anesthesia (intraperitoneal injection with 1% pentobarbital sodium, 50 mg/Kg). To ensure the welfare of the animals, our method of euthanasia followed the AVMA Guidelines for the Euthanasia of Animals.

### Specimen preparation for light microscopy

After FD for 8 weeks, the eyeballs were enucleated after euthanasia (intraperitoneal injection with pentobarbital sodium, 150 mg/Kg). Five eyeballs of each group were removed with the optic nerve 1 to 2 mm long and fixed in 4% paraformaldehyde (Solarbio, Beijing, China). The eyecups were made by cutting the eyeball along the limbus and removing the anterior segment, and then, the eyecups were fixed again in 4% paraformaldehyde at 4 °C for 24 h. After gradient alcohol dehydration, xylene transparency and paraffin machine embedding, the sclera and retina were continuously sectioned along the longitudinal axis. The sections had a thickness of 4 μm and were stained with haematoxylin-eosin (HE). A microphotographic system (OLYMPUS BH-2, Japan) was used to observe the histology and morphology of the sclera and retina.

### Specimen preparation for electron microscopy

Five eyeballs in each group were fixed in 2.5% glutaraldehyde solution (Solarbio, Beijing) at 4 °C for 72 h. First, the sclera and retina were sliced to a size of 2 mm × 2 mm size and washed three times with phosphate buffer (PBS) for 10 min each time. Second, the eyeball samples were fixed in 1% citric acid for two hours and rinsed twice with double distilled water for 10 min each time. Third, ethanol acetone was used for stepwise dehydration. Fourth, the samples were infiltrated and embedded with Epon812 epoxy resin. Then, the slice was cut into a 10 nm sample and double stained with uranyl acetate and lead citrate. Finally, a Hitachi H-600 transmission electron microscope was used to observe the sclera and retina ultrastructure and take photos.

### Colorimetric detection of the MDA content and SOD activity

After FD for 8 weeks, ten guinea pigs in each group were selected, and then, the eyeballs were removed after the same administration of euthanasia described above. After removal of the anterior segment of the eye on an ice cube, the sclera and retinal/choroidal unity were peeled off with a microscopic tweezer and placed into a cryotube. The wet weight was weighed with a precision analytical balance, and double-distilled water was added to generate a homogenate. The homogenate was centrifuged at 3000 r/min for 5 min, and the supernatant was taken for testing. According to the SOD vitality test kit instructions, we used a 96-well plate to set the sample well and the blank control well. We then added the sample to be tested and other various solutions in turn, and we added the reaction start working solution, mixed it well, and incubated it at 37 °C for 30 min. We measured the absorbance at 450 nm and calculated the SOD vitality according to the formula in the instruction manual. Based on the MDA kit standard tube absorbance, we used 0.1 ml as the standard. The standard tube absorbance minus the standard blank tube absorbance was 0.103–0.112. We calculated the MDA content according to the formula in the manual.

### Statistical analysis

SPSS statistics 22.0 software was used to analyse the results. Data are shown as the mean ± standard deviation (^−^x ± s). Comparisons between the blank control and model group were performed using independent *t* tests. Comparisons between Group II and Group III were performed using paired sample *t* tests. A value of < 0.05 was considered statistically significant between the groups.

## Results

### Changes in the refractive status of the Guinea pig eyeballs

The guinea pigs were born with hyperopia. The refractive errors of the 3-week-old guinea pigs were approximately + 3.50 D. Table 1 shows that there was no significant difference in the refractive errors among the groups before FD (*P* > 0.05). After the animals received FD for 8 weeks in the right eyes, the refractive errors of Group II were significantly increased compared with those of Group I (*P* < 0.05). The refractive errors of Group II were higher than those of Group III, and the differences were statistically significant (*P* < 0.05).

**Table 1:** Changes of refractive errors at three and 11 weeks of age in each group of guinea pigs

	Group I (*n* = 20)	Group II (n = 20)	Group III (n = 20)	*P* Values among 3 groups	*P* values of post hoc Comparison		
					I vs. II	II vs. III	I vs. III
Refractive errors, D (3-week-old guinea pigs)	+ 3.65 ± 0.27	+ 3.59 ± 0.33	+ 3.61 ± 0.29	0.8352			
Refractive errors, D (11-week-old guinea pigs)	+ 0.89 ± 0.32	−7.96 ± 0.55	−5.55 ± 0.49	<.0001	<.0001	<.0001	<.0001

Ultrasonometry A measurement results and statistics

After FD, the vitreous cavity of Group II was deepened, and the axial length of the eye increased. Compared with Group I, the difference was statistically significant (*P* < 0.01). The depth of the vitreous cavity and the axis of the right eye in the guinea pigs in Group III were compared. The length was less than that in Group II, and the difference was also statistically significant (*P* = 0.000). There was no significant difference in the depth of the anterior chamber and the thickness of the lens among the groups before and after FD (*P* > 0.05; Table 2).

**Table 2:** Changes in measurement parameters of ultrasonometry A at three and 11 weeks of age in each group of guinea pigs

	Group I (n = 20)	Group II (n = 20)	Group III (n = 20)	*P* Values among 3 groups	*P* values of post hoc Comparison		
					I vs. II	II vs. III	I vs. III
AC, mm (3-week-old guinea pigs)	1.22 ± 0.15	1.24 ± 0.06	1.23 ± 0.34	0.3216	NS	NS	NS
AC, mm (11-week-old guinea pigs)	1.31 ± 0.14	1.39 ± 0.24	1.31 ± 0.41	0.2130	NS	NS	NS
L, mm (3-week-old guinea pigs)	3.05 ± 0.15	3.05 ± 0.31	3.03 ± 0.28	0.1356	NS	NS	NS
L, mm (11-week-old guinea pigs)	3.11 ± 0.21	3.14 ± 0.16	3.12 ± 0.17	0.2031	NS	NS	NS
VC, mm (3-week-old guinea pigs)	3.42 ± 0.13	3.43 ± 0.24	3.44 ± 0.16	0.0645	NS	NS	NS
VC, mm (11-week-old guinea pigs)	3.71 ± 0.23	4.12 ± 0.13	3.97 ± 0.03	0.0046	0.0029	0.0036	0.0014
AL, mm (3-week-old guinea pigs)	7.26 ± 0.42	7.27 ± 0.16	7.27 ± 0.62	0.1124	NS	NS	NS
AL, mm (11-week-old guinea pigs)	7.95 ± 0.37	8.93 ± 0.22	8.26 ± 0.15	0.0102	0.0013	0.0002	0.0023

*NS* no significance

### Observation of the Guinea pig retinas under a light microscope

**Fig. 1:**
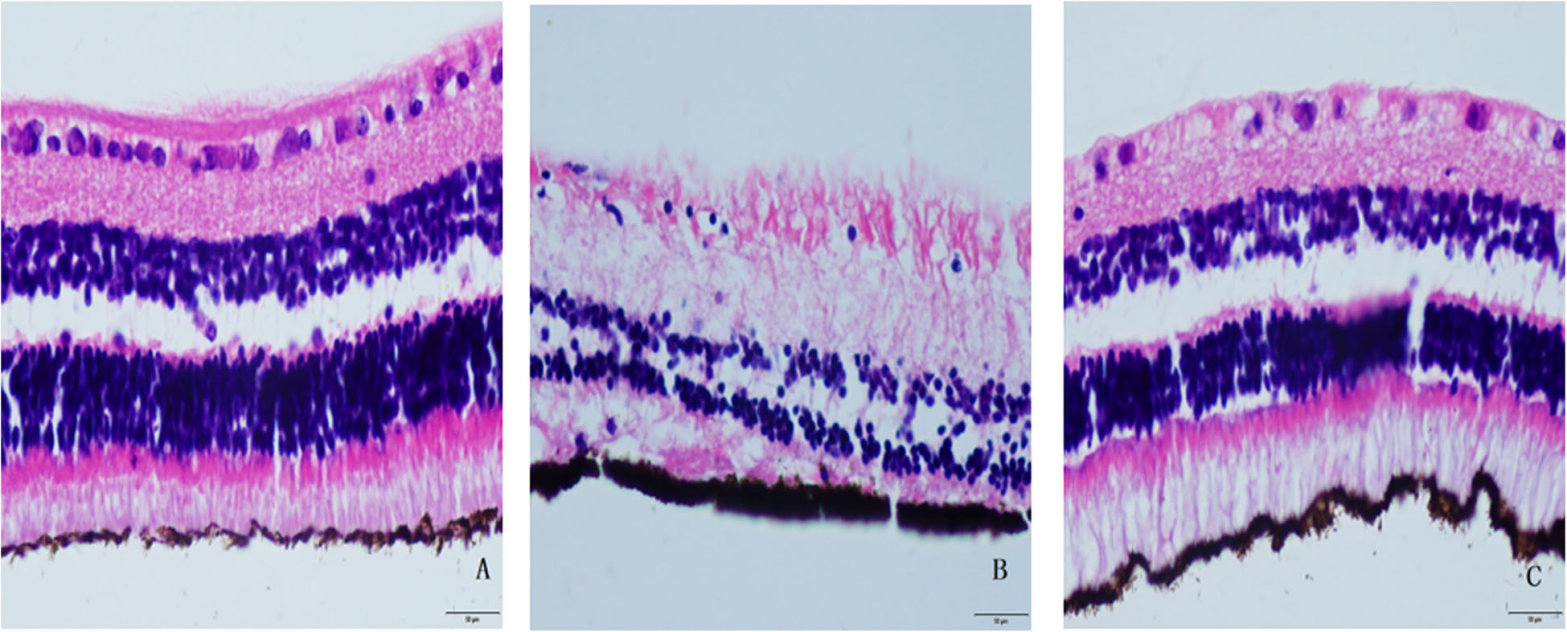
Observation of guinea pig retinas under light microscope (HE× 400)

Figure 1 showed that the retinal structure of Group I was clear, the thickness of the ganglion cell layer was normal, the monolayer was normally distributed, the nucleus was round, the nucleolus was clear and neatly arranged, there was no cell loss or deformation, and the retinal pigment epithelium (RPE) included visual cells. The outer segment protruded as small protrusions arranged in a brush-like shape and was neat and dense (Fig. 1a). After FD for 8 weeks, the retinas of Group II showed thinning, the ganglion cell and inner and outer nuclear layers were all reduced, the nucleus was small and uneven, and the cells were arranged in a disorderly manner. The microvilli protruding from the RPE layer were shorter and even showed fusion and breakage (Fig. 1b). The retinas of Group III showed only a decrease in ganglion cells, a small nucleus, and clear structures in the remaining layers (Fig. 1c).

Observation of the guinea pig retinas under an electron microscope.

**Fig. 2:**
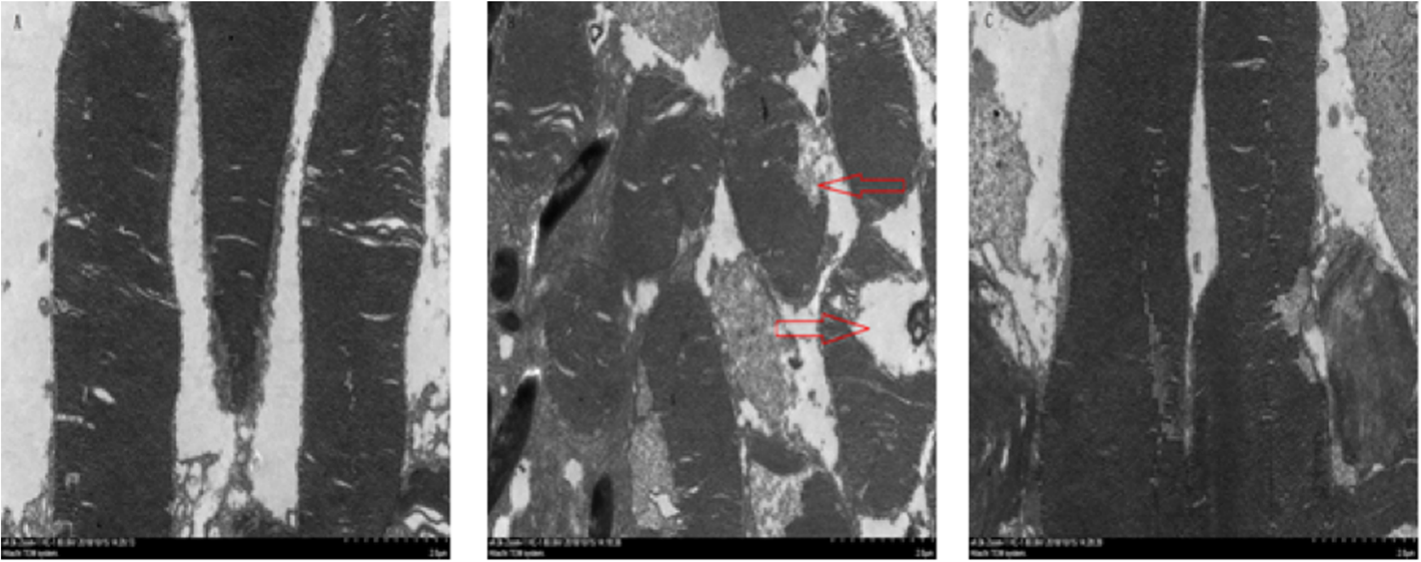
Observation of guinea pig retinal membrane disk under electron microscopic (× 4000)

**Fig. 3:**
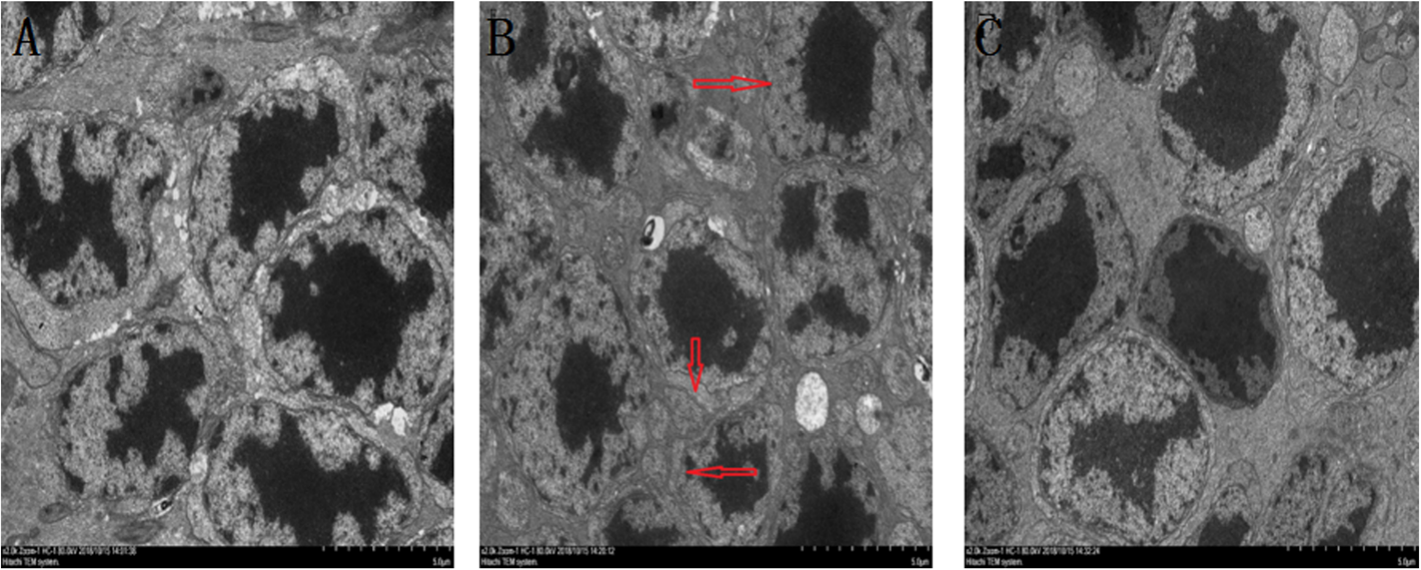
Observation of guinea pig retinal inner nuclear layers under electron microscopic (× 4000)

Figure 2 showed that under an electron microscope, the retinal disks of Group I were arranged neatly and tightly (Fig. 2a). Compared with those Group I, the membrane disks of Group II showed swelling and deformation (as shown by the red arrow in Fig. 2b). Figure 3 showed that the nuclear layer of the cell membranes was smooth and completed with uniform chromatin (Fig. 3a). Compared with Group I, Group II showed irregular contraction of the inner and outer nuclear layers and RPE cell membrane, irregular accumulation of nuclear chromatin, mitochondrial swelling, deformation and vacuolization (as shown by the red arrow in Fig. 3b). The pathological damage to the retinal membrane disk and inner nuclear layers in Group III was weaker than that in Group II (Fig. 2c, Fig. 3c).

### Observation of the Guinea pig sclera under a light microscope

**Fig. 4:**
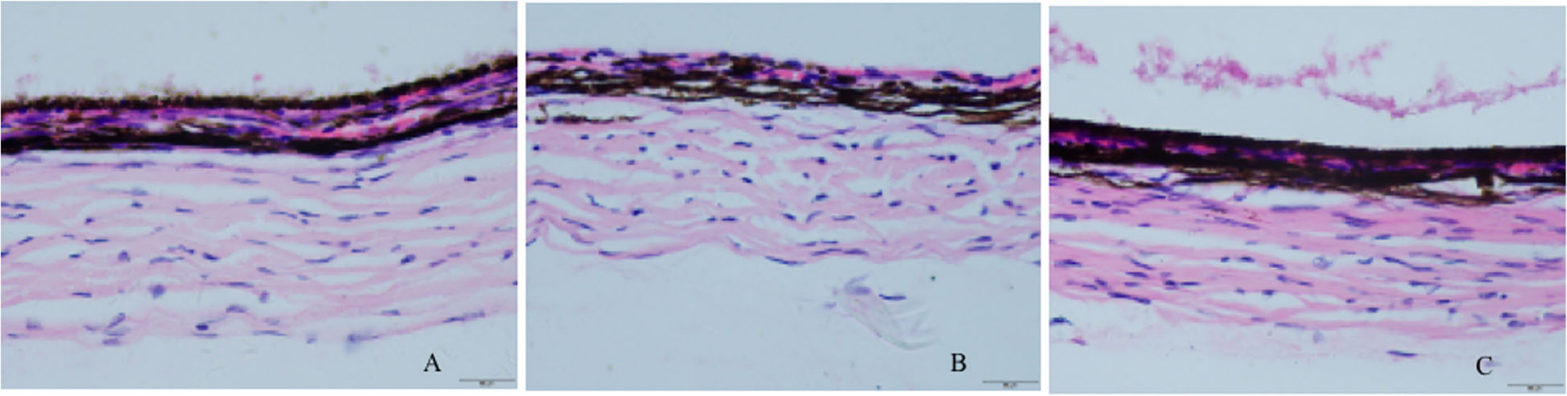
Observation of guinea pig sclera under a light microscope (HE× 400)

Figure 4 showed that the scleral thickness of Group I was normal. The fibroblasts were stained blue-purple and were fusiform or oblong, the extracellular matrix was pink, the collagen fibres were neatly arranged, and the diameters were uniform (Fig. 4a). The sclera of the guinea pigs in Group II showed obvious thinning; the fibroblast cell nuclei were distorted; the collagen fibres were sparsely distributed; the diameter was obviously reduced; the arrangement was disordered, twisted, broken, and separated; the interfibre space was increased; and the extracellular matrix was increased (Fig. 4b). The scleral thickness of Group III showed slight thinning, but the morphology was not significantly abnormal (Fig. 4c).

### Observation of the Guinea pig sclera under an electron microscope

**Fig. 5:**
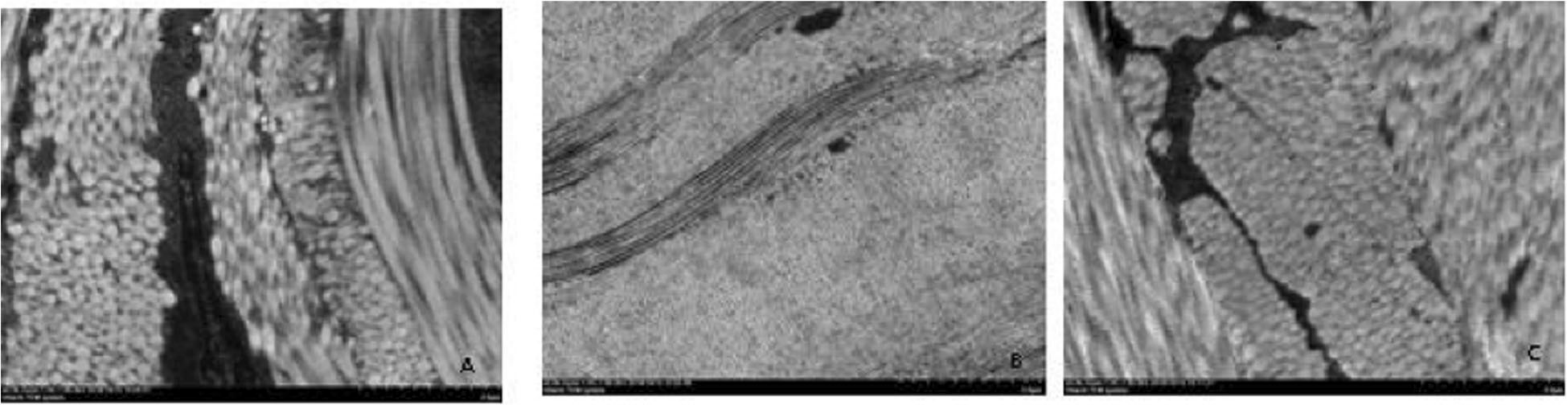
Observation of guinea pig sclera under an electron microscope (× 4000)

Figure 5 showed that the scleral tissue was composed of fibroblasts and collagen bundles parallel to the wall of the eyeball. The collagen fibres constitute the framework of the scleral tissue. The cross-sectional diameter of the collagen fibres was observed. There was no significant difference between the sclera of Group I and Group III. Compared with those of the two groups, the diameter of the FDHM was significantly reduced, and the fibre density was decreased in Group II.

The MDA content and SOD activity in the retinal/choroidal unity and sclera of the guinea pigs.

The statistical results showed that the activity of SOD and MDA, the main products of lipid peroxide, had an opposite trend. That is, under normal circumstances, the SOD of the retinal/choroidal unity and sclera remained high in the normal group, while MDA was at a low level. However, after FD, compared with that in Group I, the content of SOD in the retinal/choroidal unity and sclera of the guinea pigs in Group II decreased, while the content of MDA increased (*P* < 0.01). There was no significant difference compared with Group III (*P* > 0.05; Tables 3, 4).

**Table 3:** Content of SOD and MDA in retinal/choroidal unity of guinea pigs (*n* = 5,^−^x ± s)

Group	SOD (μg/g)	MDA (μg/l)
I	230.01 ± 14.97	4.33 ± 0.43
II	173.35 ± 29.69^ab^	9.23 ± 0.85^ab^
III	187.73 ± 18.31	8.70 ± 1.59

Compared with Group I, ^a^*P* < 0.01, compared with Group III, ^b^*P* > 0.05

**Table 4:** Content of SOD and MDA content in sclera of guinea pigs (n = 5,^−^x ± s)

Group	SOD (μg/g)	MDA (μg/l)
I	261.60 ± 2.30	0.44 ± 2.37
II	197.60 ± 2.40^ab^	1.99 ± 1.73^ab^
III	227.99 ± 4.54	1.01 ± 2.40

Compared with Group I, ^a^*P* < 0.01, compared with Group III, ^b^*P* > 0.05

## Discussion

Since the refractive status and orthotopic mechanism of guinea pigs are similar to those of humans, guinea pigs are born with hyperopia. In 3 weeks, the axial length of the guinea pigs grew rapidly, and the refractive errors decreased. The refractive state of 11-week-old guinea pigs was stabilized in the mild hyperopic eye. The FDM model has been widely used in experimental research on myopia [25, 26]. Studies have shown that guinea pigs have the characteristic of high sensitivity to FD and become ideal animal models of experimental myopia [27]. In this study, 3-week-old guinea pigs were selected as a myopic animal model. At this time, guinea pigs were in a critical period of visual development and were sensitive to the model manipulations. By prolonging the time of FD, we aimed to cause high myopia [28]. The retina is a tissue with a high oxygen demand, strong metabolism and active redox reaction. Under physiological conditions, intraocular tissues, such as the retina, are continuously stimulated by external light to cause photooxidation, which promotes the formation of oxygen free radicals. Furthermore, effective antioxidants, such as SOD, in the cells can promptly scavenge oxygen free radicals. The formation and degradation are in a dynamic equilibrium, which provides protection for tissues such as the retina and sclera. Under pathological conditions, excessive generation of oxygen free radicals or an insufficient antioxidant capacity of the body can trigger lipid peroxidation, and the main metabolite, MDA, causes damage to cells and tissues. OS can lead to retinal damage under hypoxic circumstances [29, 30]. This could be used to explain the relationship between OS and FDHM since hypoxic situations would exist chronically. The MDA content reflects the degree of attack on oxygen by oxidative cells. SOD is an important antioxidant enzyme in the body, and its activity reflects the body’s ability to scavenge oxygen free radicals [31, 32].

The results of this study showed that after 8 weeks of FD due to guinea pig eyelid suture, the myopic refractive errors decreased, with the highest value at − 8.00 D refraction, and the biometric data determined by Ultrasonometry A increased; the vitreous cavity was elongated and the axial length became longer. Compared with Group I, the difference was statistically significant (*P* < 0.01), which indicated that the high myopia model was successful. The results are trustworthy since the refractive errors were examined by a skilled optometrist and repeated three times to obtain the average value. The guinea pig model was first presented by McFadden and Wallman at ARVO in 1995. It has been reported that guinea pigs could develop deprivation myopia after a short period of time usually a few days [33]. The guinea pig model is the most common animal model for myopia in Asian laboratories [16]. As shown in a myopic animal model, there exists an approximately 3,00 D difference between the model group and the self-controlled group after occlusion for 14 days [34]. There may be species differences between guinea pigs and tree shrews. The specific mechanism for these differences needs further investigation. There was no significant difference in the biochemical parameters between Group II and the Group III (both *P* > 0.05). Deprivation in Group II did not interfere with the natural development of the contralateral eye, which is consistent with the findings of other researchers, such as Zhao [35]. Yang et al. [36] detected retinal metabolic changes in guinea pigs induced by form deprivation myopia. Although these researchers did not directly measure the SOD and MDA levels, the results they reported represented the retinal metabolite levels. That is, no statistically significant differences were revealed between the FD and self-controlled eyes. In our study, the SOD and MDA contents decreased and increased, respectively, compared with those of Group I. There are several potential possibilities to account for the mismatch with the refraction and biometric parameters. Myopia development is a time-dependent process. Thus, it is necessary to measure biochemical parameters at more time-points in further studies. We expect that these differences in biometric parameters would be significant if the sample size was larger.

## Conclusions

In summary, this experiment produced a HM status in the guinea pigs FD for 8 weeks. We observed retinal and scleral morphology, refractive errors, vitreous cavity depth, axial length change and SOD and MDA expression. Disorders in the oxygen free radical level may be involved in the development of FDHM. The results of this study provide new ideas for the pathogenesis and prevention of HM. However, the specific mechanism of action is not fully understood, and further research is needed.

## Data Availability

The datasets used and/or analysed during the current study are available from the corresponding author on reasonable request at any time.

## Abbreviations

HM: High myopia
PM: Pathological myopia
FDM: Form deprivation myopia
FD: Form deprivation
MDA: Malondialdehyde
SOD: Superoxide dismutase
FDHM: Form deprivation high myopia
AC: Anterior chamber depth
L: Lens thickness
V: Vitreous chamber depth
AL: Axial length
HE: Haematoxylin-eosin
PBS: Phosphate buffer
RPE: Retinal pigment epithelium
